# Recurrent Somatic *MAP2K1* Mutations in Papillary Thyroid Cancer and Colorectal Cancer

**DOI:** 10.3389/fonc.2021.670423

**Published:** 2021-05-11

**Authors:** Rong Bu, Abdul K. Siraj, Tariq Masoodi, Sandeep Kumar Parvathareddy, Kaleem Iqbal, Maha Al-Rasheed, Wael Haqawi, Mark Diaz, Ingrid G. Victoria, Saud M. Aldughaither, Saif S. Al-Sobhi, Fouad Al-Dayel, Khawla S. Al-Kuraya

**Affiliations:** ^1^ Human Cancer Genomic Research, Research Center, King Faisal Specialist Hospital and Research Center, Riyadh, Saudi Arabia; ^2^ Department of Surgery, King Faisal Specialist Hospital and Research Center, Riyadh, Saudi Arabia; ^3^ Department of Pathology, King Faisal Specialist Hospital and Research Centre, Riyadh, Saudi Arabia

**Keywords:** mutation, *MAP2K1*, papillary thyroid cancer, colorectal cancer, somatic

## Abstract

*Mitogen-activated protein kinase kinase 1 (MAP2K1)* is a dual specificity protein kinase that phosphorylates both threonine and tyrosine residues in *ERK*. *MAP2K1* mutations have been identified in several cancers. However, their role in Middle Eastern papillary thyroid cancer (PTC) and colorectal cancer (CRC) is lacking. In this study, we evaluated the prevalence of *MAP2K1* mutations in a large cohort of Middle Eastern PTC and CRC using whole-exome and Sanger sequencing technology. In the discovery cohort of 100 PTC and 100 CRC cases (comprising 50 *MAPK* mutant and 50 *MAPK* wildtype cases each), we found one *MAP2K1* mutation each in PTC and CRC, both of which were *MAPK* wildtype. We further analyzed 286 PTC and 289 CRC *MAPK* wildtype cases and found three *MAP2K1* mutant PTC cases and two *MAP2K1* mutant CRC cases. Thus, the overall prevalence of *MAP2K1* mutation in *MAPK* wildtype cases was 1.1% (4/336) in PTC and 0.9% (3/339) in CRC. Histopathologically, three of the four *MAP2K1* mutant PTC cases were follicular variant and all four tumors were unifocal with absence of extra-thyroidal extension. All the three CRC cases harboring *MAP2K1* mutation were of older age (> 50 years) and had moderately differentiated stage II/III tumors located in the left colon. In conclusion, this is the first comprehensive report of *MAP2K1* somatic mutations prevalence in PTC and CRC from this ethnicity. The mutually exclusive nature of *MAP2K1* and *MAPK* mutations suggests that each of these mutation may function as an initiating mutation driving tumorigenesis through *MAPK* signaling pathway.

## Introduction

*Mitogen-activated protein kinase (MAPK)* pathway has been known to play an important role in the pathogenesis and survival of many tumors, especially thyroid and colorectal cancers (1–4). Genetic alterations that aberrantly activate this kinase pathway in cancers are typically the result of *BRAF* or *KRAS* mutations in the majority of cancers displaying *MAPK* activation (5–7). It is known that *BRAF* is not the only downstream kinase in the *MAPK* pathway subject to mutation and subsequent *MAPK* activation. Several reports have identified oncogenic mutations in *MAPK kinase 1 (MAP2K1*, also called *MEK1*) as alternative mechanism for *MAPK* pathway activation in *BRAF* wild-type tumor in various cancers (8–11). Mutations in *MAP2K1*, a primary downstream effector of *RAF* kinases, are uncommon and subsequently tumors harboring these mutations need to be explored, especially in patients who might be treated with *MEK* and *RAF* targeted therapy.

*MAP2K1* encodes a dual specific serine/threonine and tyrosine kinase, activated in response to phosphorylation by *RAF* kinase (12). *MAP2K1* mutations and deletions in the activation segment have been shown to constitutively activate the protein (13, 14). Mutations of *MAP2K1* have been reported in several human cancers, especially melanoma, Langerhans histocytosis, hairy cell leukemia and lung adenocarcinoma (9–11, 15, 16). Most of the reported *MAP2K1* mutations tend to be mutually exclusive with other *MAPK* driver mutations (8, 9, 15). Mutations affecting the N-terminal negative regulatory domain encoded by exon 2 and the catalytic core encoded by exon 3 accounted for majority of previously reported *MAP2K1* mutations (9, 17–19).

Despite the major role of *MAPK* pathway in colorectal cancer (CRC) and papillary thyroid cancer (PTC), there are few reports about the prevalence of *MAP2K1* mutations in these tumor sites (17, 20–22). More importantly, data on *MAP2K1* mutations in PTC and CRC from Arab Middle Eastern ethnicity is lacking. Therefore, we sought to determine the prevalence of *MAP2K1* mutations in a large cohort of Middle Eastern PTC and CRC. In the discovery cohort, we performed whole-exome sequencing on a discovery cohort of 100 PTC samples and 100 CRC samples. We then validated our findings in set of additional 286 PTC and 289 CRC using Sanger sequencing analysis.

## Materials and Methods

### Patient Selection and Tumor Samples

The initial discovery cohort included 100 cases each of PTC and CRC (50 *MAPK* (*BRAF/KRAS/NRAS/HRAS*) mutant and 50 *MAPK* wildtype) diagnosed at King Faisal Specialist Hospital and Research Centre (KFSHRC). Subsequent validation cohort consisted of 286 PTC and 289 CRC samples, all of which lacked mutations in the *MAPK* genes. Clinico-pathological data were collected from case records, the details of which are summarized in **Tables 1** and**2**. Institutional Review Board of KFSHRC provided ethical approval for the current study. Research Advisory Council (RAC) granted waiver of informed consent for use of retrospective patient case data and archival tissue samples under project RAC# 2110 031 and 2190 016.

**Table 1:** Clinico-pathological variables for the papillary thyroid cancer patient cohort.

Clinico-pathological variables	Testing cohort (n = 100)	Validation cohort (n = 286)
	n (%)	n (%)
**Age (years)**		
Median	39.2	30.9
Range	12–74	6–89
<55	86 (86.0)	257 (89.9)
≥55	14 (14.0)	29 (10.1)
**Gender**		
Female	78 (78.0)	213 (74.5)
Male	22 (22.0)	73 (25.5)
**Histopathology**		
Classical Variant	47 (47.0)	187 (65.4)
Follicular Variant	23 (23.0)	69 (24.1)
Tall Cell Variant	15 (15.0)	8 (2.8)
Others	15 (15.0)	22 (7.7)
**Extra Thyroidal Extension**		
Absent	56 (56.0)	185 (64.7)
Present	44 (44.0)	101 (35.3)
**pT**		
T1	23 (23.0)	82 (28.7)
T2	23 (23.0)	73 (25.5)
T3	46 (46.0)	105 (36.7)
T4	8 (8.0)	26 (9.1)
**pN**		
N0	48 (48.0)	119 (41.6)
N1	40 (40.0)	142 (49.7)
Nx	12 (12.0)	25 (8.7)
**pM**		
M0	95 (95.0)	264 (92.3)
M1	5 (5.0)	22 (7.7)
**Stage**		
I	89 (89.0)	248 (86.8)
II	5 (5.0)	23 (8.1)
III	2 (2.0)	5 (1.7)
IVA	0	1 (0.3)
IVB	3 (3.0)	6 (2.1)
Unknown	1 (1.0)	3 (1.0)

**Table 2:** Clinico-pathological variables for the colorectal cancer patient cohort.

Clinico-pathological variables	Testing cohort (n = 100)	Validation cohort (n = 289)
	n (%)	n (%)
**Age (years)**		
Median	52.1	56.0
Range	13–90	22–91
≤50	46 (46.0)	96 (33.2)
>50	54 (54.0)	193 (66.8)
**Gender**		
Male	50 (50.0)	167 (57.8)
Female	50 (50.0)	122 (42.2)
**Histological subtype**		
Adenocarcinoma	86 (86.0)	261 (90.3)
Mucinous carcinoma	14 (14.0)	28 (9.7)
**Histological grade**		
Well differentiated	9 (9.0)	19 (6.6)
Moderately differentiated	72 (72.0)	237 (82.0)
Poorly differentiated	15 (15.0)	27 (9.3)
Unknown	4 (4.0)	6 (2.1)
**Tumor site**		
Left	67 (67.0)	246 (85.1)
Right	33 (33.0)	43 (14.9)
**pT**		
T1	3 (3.0)	8 (2.8)
T2	8 (8.0)	50 (17.3)
T3	70 (70.0)	192 (66.4)
T4	15 (15.0)	36 (12.5)
Unknown	4 (4.0)	3 (1.0)
**pN**		
N0	44 (44.0)	147 (50.9)
N1	29 (29.0)	91 (31.5)
N2	22 (22.0)	48 (16.6)
Nx	5 (5.0)	3 (1.0)
**pM**		
M0	72 (72.0)	257 (88.9)
M1	28 (28.0)	32 (11.1)
**TNM Stage**		
I	9 (9.0)	44 (15.2)
II	32 (32.0)	97 (33.6)
III	30 (30.0)	116 (40.1)
IV	28 (28.0)	32 (11.1)
Unknown	1 (1.0)	0

### DNA Isolation

DNA samples were extracted from formalin-fixed and paraffin-embedded (FFPE) CRC tumor tissues utilizing Gentra DNA Isolation Kit (Gentra, Minneapolis, MN, USA) according to the manufacturer’s protocols as elaborated in the previous studies (23).

### Whole-Exome Sequencing Analysis

DNA samples were analyzed by whole exome sequencing using Illumina Novaseq. Sequencing reads in fastq format were mapped to the human genome version 19 using Burrows-Wheeler Aligner (BWA) (24). PCR duplicate marking, local realignment and base-quality recalibration were performed with Picard tools (http://broadinstitute.github.io/picard/) and GATK (25).

Single nucleotide variants (SNVs) and indels were called with MuTect (26), and VarScan2 (http://varscan.sourceforge.net) respectively. Annotation of somatic variants was performed using ANNOVAR (27). The SNVs that passed the standard Mutect and VarScan2 filters were retained, and common SNPs with minor allele frequency (MAF) of > 0.001 in dbSNP, the NHLBI exome sequencing project, 1000 Genomes and our in-house exome database of around 800 normals were removed for further analysis. Somatic SNVs were manually checked using Integrated Genomics Viewer (IGV) to filter out the artifacts.

### Sanger Sequencing Analysis

Sanger sequencing technology was utilized to sequence entire coding and splicing regions of exons 2 and 3 in *MAP2K1* gene among 286 *MAPK* wildtype PTC and 289 MAPK wildtype CRC samples as validation cohort. In addition, the pathogenic variants detected by Exome sequencing analysis were further confirmed by Sanger sequencing analysis. Primer 3 online software was utilized to design the primers (available upon request). PCR and Sanger sequencing analysis were carried out as described previously (28). Reference sequences were downloaded from the NCBI GenBank and sequencing results were compared with the reference sequences by Mutation Surveyor V4.04 (Soft Genetics, LLC, State College, PA).

### Pathogenicity Assessment of Mutations

Mutations were characterized as pathogenic according to The American College of Medical Genetics and Genomics (ACMG) guidelines or found functional in The Clinical Knowledgebase database (https://ckb.jax.org/). The remaining mutations were termed pathogenic if found deleterious by two of the three in silico algorithms (SIFT, PolyPhen, and MutationTaster).

## Results

### *MAP2K1* Mutation in PTC and CRC

**Figure 1:**
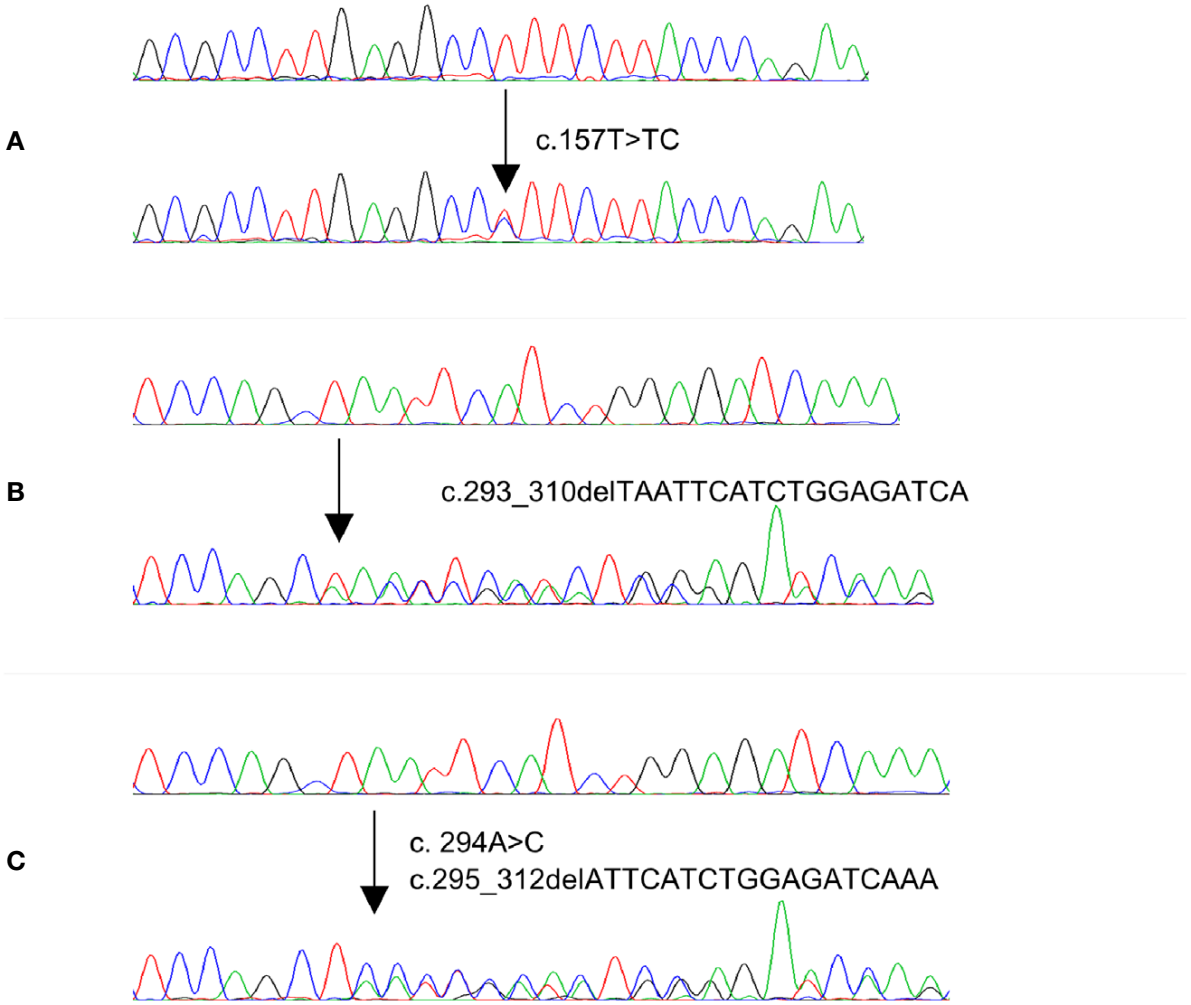
Electropherogram of three *MAP2K1* representative mutations identified in PTC and CRC cases. Upper traces represent normal sequences while lower traces show mutated sequences.

In the discovery cohort of PTC cases consisting of 50 *MAPK* wildtype and 50 *MAPK* mutant cases, one inframe deletion (p.I99_K104del) of *MAP2K1* was identified in a *MAPK* wildtype case, accounting for 2% (1/50) of all *MAPK* wildtype PTC cases. No mutation was detected in *MAPK* mutant PTC cases. This mutation was reported as gain of function mutation by The Clinical Knowledgebase (CKB, https://ckb.jax.org). Furthermore, in the discovery cohort of CRC cases, one missense mutation, p.93V>A, was detected in one (2%) of 50 *MAPK* wildtype cases, while no mutation was found in 50 *MAPK* mutant cases. This mutation was predicted as pathogenic mutation by SIFT and MutationTaster (**Table 3** and **Figure 1**).

**Table 3:** *MAP2K1* mutations in papillary thyroid cancer and colorectal cancer cases.

S. No.	Mutation	Exon	Organ Site	No. of cases	SIFT	PolyPhen	Mutation Taster
1	c.294A>C & c.295_312delATTCATCTGGAGATCAAA; p.I99_K104del	Ex3	PTC	1	N/A	N/A	N/A
2	c.293_310delTAATTCATCTGGAGATCA; p.I99_K104del & p.L98Q	Ex3	PTC	2	N/A	N/A	N/A
3	c.302_307delTGGAGA;p.E102_I103del	Ex3	PTC & CRC	2	N/A	N/A	N/A
4	c.278T>C;p.93V>A	Ex2	CRC	1	Damaging	Benign	Disease causing
5	c.157T>TC;p.53F>F/L	Ex2	CRC	1	Tolerated	Probably damaging	Disease causing

N/A, not available.

Since *MAP2K1* mutations were only identified in *MAPK* wildtype cases, we further analyzed 286 *MAPK* wildtype PTC and 289 *MAPK* wildtype CRC cases as validation cohort. Among 286 PTC cases, three cases (1.0%) carried gain of function mutations, while in 289 CRC cases, two (0.7%) cases harbored gain of function mutations of *MAP2K1* gene. Altogether, four out of 336 (1.1%) *MAPK* wildtype PTC cases carried gain of function mutations while three out of 339 (0.9%) *MAPK* wildtype CRC cases carried mutations (**Table 3** and **Figure 1**). Among five mutations identified in our study, one was novel mutation while four were reported as somatic mutations in COSMIC (**Supplementary Table 1**).

### Clinico-Pathological Characteristics of *MAP2K1* Mutant PTC and CRC Cases

Among the four PTC cases harboring *MAP2K1* mutation, the median age was 51 years (range: 30–70 years). The mutations were distributed equally among male and female PTC patients (two each). 75% (3/4) of PTCs with *MAP2K1* mutation were of follicular variant and 25% (1/4) were classical variant. 75% (3/4) of the tumors harboring *MAP2K1* mutation were encapsulated with only one case showing tumor capsule invasion. None of the cases showed vascular invasion or extra-thyroidal extension. All the tumors were unifocal. 75% (3/4) of patients had stage I PTC and one patient had stage IV with distant metastasis to the brain. On follow-up (median: 5.5 years, range: 1–9 years), the patient with distant metastasis died due to disease progression, whereas the other three patients had no evidence of disease (**Table 4**).

**Table 4:** Clinico-pathological characteristics of *MAP2K1* mutant papillary thyroid cancer case.

Case no.	Cohort	Age (years)	Gender	Histopathologic subtype	Encapsulation	Vascular Invasion	Extra-thyroidal extension	Focality	pT	pN	pM	Stage	Status
1	Testing	70	Female	Follicular variant	No	Negative	Absent	Unifocal	T3a	N0	M1	IV-B	Deceased (due to metastatic disease)
2	Validation	30	Male	Follicular variant	Yes	Negative	Absent	Unifocal	T2	N0	M0	I	No evidence of disease
3	Validation	50	Male	Follicular variant	Yes	Negative	Absent	Unifocal	T1	N0	M0	I	No evidence of disease
4	Validation	52	Female	Classical variant	Yes	Negative	Absent	Unifocal	T1	N0	M0	I	No evidence of disease

Among the three CRC cases harboring *MAP2K1* mutation, the median age was 79 years (range: 60–81 years). All patients were male. All three patients had moderately differentiated left sided tumors. One patient presented with stage II disease and two patients presented with stage III disease. One of the tumors showed mismatch repair deficiency by immunohistochemistry (**Table 5**).

**Table 5:** Clinico-pathological characteristics of *MAP2K1* mutant colorectal cancer cases.

Case no.	Cohort	Age (years)	Gender	pT	pN	pM	Stage	Grade	Tumor site	MMR IHC
1	Testing	60	Male	T3	N1	M0	III	Grade 2	Left colon	dMMR
2	Validation	81	Male	T3	N2	M0	III	Grade 2	Left colon	pMMR
3	Validation	79	Male	T3	N0	M0	II	Grade 2	Left colon	pMMR

dMMR, deficient mismatch repair; pMMR, proficient mismatch repair.

## Discussion

Targeted therapies have emerged as a promising cancer therapeutic option due to their effectiveness in treating “oncogene-addicted” cancers (29, 30). Therefore, accurate prediction of anti-tumor effects of molecularly targeted agents before clinical trial design and implementation in cancer patients is important to achieve the goal of precision medicine. Many studies investigating the effect of inhibitors of the *MAPK* pathways in thyroid and colorectal cancers highlight the importance of identifying mutations in such signaling pathway and their impacts on the subsequent efficacy of targeted therapies, thus reinforcing the importance of better personalized therapeutic strategies (31–34). In this study, we identified the prevalence of *MAP2K1* mutations in Middle Eastern PTC and CRC.

*MAP2K1* mutations have been reported in several types of tumors, including CRC and PTC (9, 11, 15, 17, 20, 21, 35). The majority of previously reported *MAP2K1* mutations or deletions targeted Exon 2 and 3 which encode the negative regulatory domain and the catalytic core (9, 17–19). Due to the recent easy access to next generation sequencing, we sought to analyze a small discovery cohort of 100 PTC and 100 CRC samples using exome sequencing to identify *MAP2K1* mutations and their correlation to other *MAPK* pathway mutations, especially since recent reports have shown mutual exclusivity between *MAP2K1* mutations and *BRAFV600E* mutations in these organ sites (17, 21). We have identified one case of inframe deletion in Exon 3 of PTC cohort and one CRC case carrying a missense mutation in Exon 2. Each of the above mentioned mutations occurred in a *BRAF* wild-type context, consistent with the notion that *BRAF* & *MAP2K1* are acting in the same transformation pathway.

We then further expanded our study to include additional 286 PTC and 289 CRC samples as validation cohort. We found overall mutations in *MAP2K1* occurring in 1.1% (4/336) of PTC and 0.9% (3/339) CRC *MAPK* wildtype cohorts. *MAP2K1* mutations have been classified into 3 classes (36). Class 1 *MAP2K1* mutations are *RAF* dependent and are least activating. Class 2 *MAP2K1* mutations are activating in nature but can be upregulated further by upstream *RAF*. Class 3 *MAP2K1* mutations lead to auto-phosphorylation of *MEK* which is independent of *RAF* and usually is mutually exclusive with other mutations that activate *MAPK* signaling and therefore considered driver mutations. Previous report has shown the ability of class 3 mutation *in vivo* to promote tumor growth more efficiently than class 1 and class 2 mutations (36).

In this study, we detected *MAP2K1* mutation in 4/336 (1.1%) PTCs that otherwise had no known *MAPK* pathway genetic alterations. All mutations identified in our PTC cohort were in-frame deletions/class 3 mutations and located in the kinase domain encoded by Exon 3. These deletions cause gain of *MAP2K1* function as demonstrated by activity, independent of *Raf* and increased phosphorylation of *Mek* and *Erk* relative to wild-type *MAP2K1 (* 37) (CKB database, https://ckb.jax.org) and have been reported previously (8, 36, 38, 39). Interestingly, the missense mutation c.157T>TC;p.53F>F/L within the negative regulatory region was also reported as gain of MAP2K1 function due to increase of Erk and Mek phosphorylation (16). Although *MAP2K1* mutated PTCs show no predilection to gender, stage and grade, majority (75%; 3/4) were encapsulated and showed follicular pattern. All of the *MAP2K1* mutated cases were unifocal and showed absence of any extra-thyroidal extension. Upon follow-up, only one adverse event was registered.

Till date, only two reports have been published about the incidence of *MAP2K1* mutations in PTC. First published report was limited to *MAP2K1* Exon 2 mutations which was not present in any of the PTC cases tested (20). Second, more recent report, examined Exon 2 and 3 and identified *MAP2K1* mutation in 2% of their 101 PTC cohort which is higher than the frequency of mutations in our cohort (1.1%), but the difference was not statistically significant (17). Similar to our results, all PTC mutations identified were limited to Exon 3 (catalytic core) and all tumors showed similar histology where all the cases harboring *MAP2K1* mutations were encapsulated, had predominantly follicular architecture and were intra-thyroidal with no lymphovascular invasion. In contrast to our study, TCGA reported *MAP2K1* mutations in only 0.2% (1/482) PTC cases (40, 41).

Furthermore, our study has shown *MAP2K1* mutation in 0.9% (3/339) of CRCs, all mutually exclusive with other *MAPK* driver mutations. One mutation was classified as Class 2 *MAP2K1* mutation and was similar to previous studies (36), whereas one Class 3 mutation (p.E102_I103del) was identified in our cohort. The Cancer Genome Atlas (TCGA) has reported *MAP2K1* mutations in a high percentage (2.4%; 13/549) of CRC cases (40, 42, 43), while other studies have shown incidence varying from 1% to 2% (20–22). All patients harboring *MAP2K1* mutation in our cohort were older than 50 years with moderately differentiated tumors. Compared to previous studies (21, 41, 44), the clinico-pathological characteristics of *MAP2K1* mutant CRC cases in our cohort differed with respect to gender distribution, tumor location and tumor stage.

The limitation of the present study is the low number of *MAP2K1* mutant cases in both PTC (4/336, 1.2%) and CRC (3/339, 0.9%) and are not adequate for performing valid statistical associations. Subsequent multicenter study is needed to identify significant associations of these variants in Middle Eastern population.

In conclusion, this is the first comprehensive report of *MAP2K1* somatic mutations prevalence in PTC and CRC from this ethnicity. The mutually exclusive nature of *MAP2K1* and *BRAF* mutations suggests that each of these mutation may function as an initiating mutation driving tumorigenesis through *MAPK* signaling pathway. This might have important clinical implications for the use of *BRAF* and *MEK* inhibitor therapies in a subset of Middle Eastern PTC and CRC patients.

## Data Availability Statement

The raw sequencing data has been deposited in Sequence Read Archive (SRA) database of National Center for Biotechnology Information (NCBI) under accession number PRJNA726736 (https://www.ncbi.nlm.nih.gov/bioproject/PRJNA726736).

## Ethics Statement

The studies involving human participants were reviewed and approved by Research Advisory Council (RAC). The ethics committee waived the requirement of written informed consent for participation.

## Author Contributions

RB, AS, and TM designed the study, performed targeted capture sequencing analysis, and helped write the manuscript. SP and KI analyzed the data. MA-R, WH, MD, IV, and SA were involved in performing the experiments. SA-S and FA-D provided the clinical resources and executed the study. KA-K designed the study, supervised the study, and drafted the manuscript. All authors contributed to the article and approved the submitted version.

## Conflict of Interest

The authors declare that the research was conducted in the absence of any commercial or financial relationships that could be construed as a potential conflict of interest.
