# Supplementary material for: Recurrent Somatic MAP2K1 Mutations in Papillary Thyroid Cancer and Colorectal Cancer
Source: Front Oncol. 2021 May 11;11:670423. doi: 10.3389/fonc.2021.670423 (PMC8144646; doi:10.3389/fonc.2021.670423)
Supplement: Supplementary file 1 [file Table_1.docx]

**Supplementary Table 1: Previously reported *MAP2K1* mutations details**

| **Mutation** | **Domain** | **CKB*** | **Activity independent of Raf** | **Increased phosphorylation of Mek** | **Increased phosphorylation of Erk** | **COSMIC** |
| --- | --- | --- | --- | --- | --- | --- |
| p.I99_K104del | Kinase | Gain of function | Yes | Yes | Yes | Yes |
| p.I99_K104del & p.L98Q | Kinase | Gain of function | Yes | Yes | Yes | Yes |
| p.E102_I103del | Kinase | Gain of function | Yes | Yes | Yes | Yes |
| p.53F>F/L | Negative regulatory | Gain of function | N/A | Yes | Yes | Yes |

CKB: Clinical Knowledge Database; N/A: Not available
